# Inference of Biochemical S-Systems via Mixed-Variable Multiobjective Evolutionary Optimization

**DOI:** 10.1155/2017/3020326

**Published:** 2017-05-21

**Authors:** Yu Chen, Dong Chen, Xiufen Zou

**Affiliations:** ^1^School of Science, Wuhan University of Technology, Wuhan, Hubei 430070, China; ^2^School of Mathematics and Statistics, Wuhan University, Wuhan, Hubei 430072, China

## Abstract

Inference of the biochemical systems (BSs) via experimental data is important for understanding how biochemical components in vivo interact with each other. However, it is not a trivial task because BSs usually function with complex and nonlinear dynamics. As a popular ordinary equation (ODE) model, the S-System describes the dynamical properties of BSs by incorporating the power rule of biochemical reactions but behaves as a challenge because it has a lot of parameters to be confirmed. This work is dedicated to proposing a general method for inference of S-Systems by experimental data, using a biobjective optimization (BOO) model and a specially mixed-variable multiobjective evolutionary algorithm (mv-MOEA). Regarding that BSs are sparse in common sense, we introduce binary variables indicating network connections to eliminate the difficulty of threshold presetting and take data fitting error and the *L*_0_-norm as two objectives to be minimized in the BOO model. Then, a selection procedure that automatically runs tradeoff between two objectives is employed to choose final inference results from the obtained nondominated solutions of the mv-MOEA. Inference results of the investigated networks demonstrate that our method can identify their dynamical properties well, although the automatic selection procedure sometimes ignores some weak connections in BSs.

## 1. Introduction

Biochemical systems (BSs) consist of many components, which interact with each other in a complex way to act as integrated dynamic systems. Inference of biochemical systems is dedicated to identify how these components interact with each other and helpful to investigate the dynamical properties of these complex systems. In the past decades, the (probabilistic) Boolean networks [1, 2], the (dynamic) Bayesian networks [3, 4], and some other probabilistic or statistical methods [5, 6] have been proposed to confirm connections between components in BSs. Although the probability- or statistics-based models can properly address the negative effects of data noise, they cannot precisely incorporate the dynamical properties of BSs. Because ordinary differential equation (ODE) models that produce directed signed graphs are not only suited for steady-state and time-series profiles but also able to work entirely in classical category [7], they are widely utilized to model various kinds of BSs [8, 9].

In biochemical system theory (BST), the S-System model incorporating the pow-law formalism is considered as an effective and consistent mathematical model to represent and analyze the biological systems [10]. Its mathematical formalism is a nonlinear ODE system:

(1) $$\begin{array}{c} \frac{dX_{i}}{dt}=\alpha_{i}\overset{N}{\underset{j=1}{\prod }}X_{j}^{g_{ij}}-\beta_{i}\overset{N}{\underset{j=1}{\prod }}X_{j}^{h_{ij}},\;i=1,\ldots ,N, \end{array}$$

where *X*_*i*_ represents the concentration of reaction component *i* and *N* is the total number of components in the investigated network. In the S-System, there are totally *N*(2*N* + 2) parameters, including the positive rate constants *α*_*i*_, *β*_*i*_ ∈ *ℝ*^+^ and the kinetic constants *g*_*i*,*j*_, *h*_*i*,j_ ∈ *ℝ*, *i*, *j* = 1,…, *N*. Although the number of parameters to be determined is relatively large for inference of S-System, it is also employed to reconstruct large-scale GRNs [11–13], attributed to its powerful approximation of dynamics of biochemical reactions.

Inference of S-Systems is available when there is a time-course experimental data set {*X*_*i*,exp_(*t*), *t* = 0,…, *T*, *i* = 1,…, *N*} of all components, implemented by minimizing the differences between experimental data and numerical results. To address the ill-posedness of this reverse problem, minimization of the differences is usually normalized and penalized as [14, 15]

(2) min errΘ=∑t=1T ∑i=1NXi,calt,Θ−Xi,exptXi,expt2+λLΘ,

where *X*_*i*,cal_(*t*, Θ) is the numerical results of *X*_*i*_ at time *t* and *λ* is the penalization parameter that is problem-dependent. To make the objective function continuous, *L*(Θ) is commonly taken as

(3) $$\begin{array}{c} L\left(\;\Theta \;\right)=\overset{N}{\underset{i=1}{\sum }}\;\overset{N}{\underset{j=1}{\sum }}\left|\;g_{i,j}\;\right|+\left|\;h_{i,j}\;\right|. \end{array}$$

Nonlinear model (2) of S-Systems has a complicated landscape, which results in the preference to solve it by evolutionary algorithms (EAs) [16, 17]. When evaluating the candidate network parameters in the population of EAs, the ODE system should be solved via some numerical method such as the Runge-Kutta method, which could lead to a computationally-heavy evaluation process. Thus, Tsai and Wang [18] used an allocation method to decouple the ODE system. However, this method introduces an allocation parameter for evaluation of candidate solutions, which is hard to debug for its dependence on the investigated problems and available data sets. Liu et al. [19] developed a separable parameter estimation method (SPEM) to decouple the S-System. But, in their method, the rate constants are numerically determined by the least square method, which could be computationally difficult because it has to compute inverse of a 2 × 2 matrix that is the product of a 2 × *N* matrix and its transpose (the computational difficulty concerns not only the time complexity but also the stability of algorithm for computation of inverse matrices).

Since inference of BSs simultaneously addresses several issues, multiobjective optimization models could be an available alternative for this problem. Liu and Wang [20] proposed a three-objective optimization model simultaneously minimizing the concentration error, slope error, and interaction error and then transformed it to a single-objective optimization problem by converting two objectives into constraints. However, transformation of the multiobjective model to the single-objective model greatly depends on a prior information on network connections of the investigated network. Koduru et al. [21] and Cai et al. [22] simultaneously minimized data error for several different data sets, but they did not try to minimize the network connections/parameter norms to get sparse networks, which makes it more difficult to set a threshold for pruning net connections. To address the defect of model (2) that *λ* has to be regulated to ensure that its global optimal solutions do lie around the true network parameters, Spieth et al. [23] took the data error and connection number as two minimization objectives and solve it using a multiobjective evolutionary algorithm. However, they did not work on how to choose an appropriate Pareto solution as the final inference result.

This work is dedicated to address the aforementioned shortcomings in existing works. To eliminate the difficulties of debugging regularization parameters in regularized methods, we construct a biobjective optimization (BOO) model that tries to simulate the dynamical properties of S-Systems by minimizing the error between computed derivatives and estimated slopes, simultaneously driving the network topology as sparse as possible. Meanwhile, fitting of derivatives also makes it possible for decoupling S-Systems without incorporation of extra parameters. For solution of the proposed BOO model, we propose a mix-variable multiobjective evolutionary algorithm (mv-MOEA) in which a candidate network configuration is represented with combination of binary variables indicating network connections and real variables of parameter values. Then, an automatic selection procedure (ASP) is employed to take the final inference results as one from the obtained nondominated solutions of BOO. Because the ASP runs tradeoff between fitting errors and network connections by locating the knee regions on the curves of normalized objective values, it can obtain a preferred sparse network configuration with the absence of a prior information on network connections.

The rest of this paper is organized as follows.  Section 2 introduces the inference method proposed in this work. Then, effectiveness of our method is validated by benchmark S-Systems in Section 3. Finally, Section 4 draws the conclusions and presents the future work.

## 2. Method

The inference method based on multiobjective evolutionary optimization (IM-MOEO) consists of three parts: the biobjective optimization (BOO) model, the mixed-variable multiobjective evolutionary algorithm (mv-MOEA), and the automatic selection procedure (ASP), which are, respectively, presented in the following.

### 2.1. The Biobjective Optimization Model

#### 2.1.1. Decoupling the S-System

To decrease number of parameters to be confirmed, the S-System is firstly decoupled before we try to infer it by experimental data. At the same time, decoupling the S-System also reduces the time complexity of evaluation process. Dynamical properties of the autonomous S-System can be fitted by approximating the derivative values of component concentration at each time point: for the *i*th equation in (1) we try to minimize the difference between two sides of each equation as

(4) min erriΘi=Si−S^i22,i=1,…,n,

where *S*_*i*_ represents the vector of *dX*_*i*_(*t*)/*dt* at all time points, approximated by the five-point numerical formula. ${\hat{S}}_{i}$, the slope vector corresponding to parameter vector Θ_*i*_, is computed via the right part of the *i*th equation for all time points [19].

#### 2.1.2. Representation of an S-System

By decoupling the S-System (1), we can infer *N* equations one by one. The *i*th equation is characterized as network connections and parameter values and represented by **x**_**i**_ = (**bx**_**i**_, **rx**_**i**_), where **bx**_**i**_ = (*bx*_*i*,1_,…, *bx*_*i*,2*N*_) and **rx**_**i**_ = (*rx*_*i*,1_,…, *rx*_*i*,2*N*+2_) are binary and real vectors, respectively. Then, the kinetic constants can be confirmed by

(5) gij=bxi,j·rxi,j,hij=bxi,N+j·rxi,N+j,

∀*j* = 1,…, *N*. Moreover, we can also get values of rate constants by *α*_*i*_ = *rx*_*i*,2*N*+1_ and *β*_*i*_ = *rx*_*i*,2*N*+2_.

#### 2.1.3. The Biobjective Optimization Model

Once the S-System is decoupled, we can infer *N* ODEs one by one. The biobjective optimization model for inference of the *i*th ODE is

(6) min erriΘi L0Θi,

where Θ_*i*_ = (*g*_*i*1_,…, *g*_*iN*_, *h*_*i*1_,…, *h*_*iN*_, *α*_*i*_, *β*_*i*_) is the parameters to be confirmed, *L*_0_(Θ_*i*_) is the *L*_0_ norm of (*g*_*i*1_,…, *g*_*iN*_, *h*_*i*1_,…, *h*_*iN*_). Minimization of *L*_0_(Θ_*i*_) is dedicated to obtain a sparse network topology. Thanks to introduction of binary variables, the *L*_0_-norm can be computed as

(7) $$\begin{array}{c} L_{0}\left(\;\Theta_{i}\;\right)=\overset{2N}{\underset{j=1}{\sum }}bx_{i,j}. \end{array}$$

So, no threshold is needed to prune the network connections.

### 2.2. The Mixed-Variable Multiobjective Evolutionary Algorithm

Because there are at most *N* connections for each component in (1), the BOO model (6) has at most *N* + 1 Pareto solutions (for an *N*-order S-System, values of *L*_0_(Θ_*i*_) are restricted in {0,1,…, *N* + 1}). So, no diversity keeping strategy is necessary to obtain a set of uniformly distributed efficient vectors if the population size is set greater than *N* + 1. Meanwhile, the BOO model includes mixed variables and mixed-objectives, which make it difficult to solve it. Thus, we propose a mixed-variable multiobjective evolutionary algorithm (mv-MOEA) for this model. The mv-MOEA employs a respective evolution strategy for discrete and binary variables, which is beneficial to both the global exploration in the mix-variable search region and the local exploitation in the real variable subregion.

When inferring an equation of an S-System of *N* components, mv-MOEA employs a population* Pop* with assistance of* OldPop* to accelerate convergence of binary search [24]. Both* Pop* and* OldPop* are of size* PopSize* and separated into the discrete section and real section as

(8) Pop=BPop,RPop,OldPop=OldBPop,OldRPop.

Considering that a solution is evaluated by combining the binary and real variables, we also use an archive *Arc* = [*BArc*, *RArc*] to save promising network topologies, as well as a *Pool*_*i*_ of real vectors for the *i*th solution in *Pop* to promote the search in feasible regions of real variables. The framework of mv-MOEA is described as follows.

**Step 1 (initialization).**

Randomly generate *Pop*, *OldPop*, and *Arc* of* PopSize* individuals and evaluate them. ∀*i* = 1,…, *PopSize*, initialize *Pool*_*i*_ to be a set of* PopSize* randomly generated (2*N* + 2)-dimensional real vectors. By combining with the *i*th binary individual in *BPop*, evaluate all vectors in *Pool*_*i*_ via (6). Randomly select an individual in *Pop* to be *gbest* = (*bgbest*, *rgbest*).

**Step 2 (recombination).**

Generate* PopSize* offspring *Off*_*i*_ = (*boff*_*i*_, *roff*_*i*_), *i* = 1,…, *PopSize* and combine them with *Pop* to construct the intermediate population *IPop* = [*BIPop*, *RIPop*].

**Step 3 (sorting).**

Sort *Pool*_*i*_, *i* = 1,…, *PopSize* via their fitting errors (the fitting error refers to the first objective of model (6)) and denote the worst one as *pool*_*i*,*w*_. Compute dominance ranks of individuals in *IPop* via the Pareto dominance relation. For individuals of the same rank, sort them in ascending order via their *L*_0_-norm (the second objective of (6)).

**Step 4 (updating).**

Set *OldPop* = *Pop* and update *Pool*_*i*_ by *Off*_*i*_, *i* = 1,…, *PopSize*. Replace *Pop* with* PopSize* best individuals in *IPop* and update *Arc* by the rest of *IPop*. *gbest* is randomly selected from *Pop* or *Arc*.

**Step 5 .**

If the stopping criterion is not satisfied, go to Step 2; otherwise, output the nondominated solutions and the iteration process ceases.

#### 2.2.1. Recombination

For *i* = 1,…, *PopSize*, *Off*_*i*_ is generated by three randomly selected parents *Parent*_1_, *Parent*_2_, and *Parent*_3_:

- With a probability *p*_1_, *Parent*_1_ and *Parent*_3_ are randomly selected from *Pop*, and *Parent*_2_ is randomly selected from *OldPop*.
- Otherwise, *Parent*_1_, *Parent*_2_, and *Parent*_3_ are randomly selected from *Arc*.

Then, the binary and real part are, respectively, generated as follows.

- The bit-string *boff*_*i*_ is generated by the bit-strings of *Parent*_1_, *Parent*_2_, and *Parent*_3_ according to the binary recombination strategy proposed in [24].
- The real vector *roff*_*i*_ is generated by the DE/rand/1 mutation and the binary crossover strategies [25]. With a probability *p*_2_, three parents are the real parts of *Parent*_1_, *Parent*_2_, and *Parent*_3_; otherwise, *roff*_*i*_ is generated by the real part of *Parent*_1_ and two real vectors randomly selected from *Pool*_*i*_.

Finally, we combine *boff*_*i*_ and *roff*_*i*_ to obtain the candidate solutions *off*_*i*_ = (*boff*_*i*_, *roff*_*i*_), *i* = 1,…, *PopSize*.

#### 2.2.2. Updating

If the fitting error of *off*_*i*_ is smaller than that corresponding to *pool*_*i*,*w*_ in *Pool*_*i*_, replace *pool*_*i*,*w*_ with *roff*_*i*_. *gbest* is selected from the population *Pop* with probability *p*_3_; otherwise, it is selected from *Arc*. Since the archive *Arc* is here adopted to guide the convergence, it is updated with respect to the hamming distances. An offspring *off*_*i*_ is employed to update *Arc* as follows.

- If the hamming distance between *broff*_*i*_ and *barc* is greater than zero for any *arc* = (*barc*, *rarc*) ∈ *Arc*, update the archive member *arc*_*w*_ = (*barc*_*w*_, *rarc*_*w*_) by *x* = (*bx*, *rx*) if *x* = (*bx*, *rx*) has a better fitting error. Here, *arc*_*w*_ is the archive member with the worst fitting error when the hamming distance between any two archive members is greater than zero; otherwise, it is the worst one of archive members that have repeated bit-string;
- otherwise, compare *broff*_*i*_ with archive members with repeated bit-strings and replace by it one with the worst fitting error.

In this work, *p*_1_, *p*_2_, and *p*_3_ are set to be 0.8, 0.7, and 0.8, respectively.

### 2.3. Evaluation of the Obtained Nondominated Solutions

The mx-MOEA generates a set of nondominated solutions of the BOO model (6) for each equation in the S-System, where each nondominated solution represents a configuration of this equation. Because two objectives of the nondominated solutions conflict with each other, all of them could be a candidate configuration of the investigated equation. Thus, selecting one from nondominated solutions needs to address a tradeoff between network connections and fitting errors. Considering that two objectives could be of different orders of magnitude, we first normalize two objectives by their maximum values. Then, two scores Score1_*i*,*k*_ and Score2_*i*,*k*_ can be obtained for the *k*th solution of equation *i*. Now, a tradeoff strategy could be employed to select one solution as the final inference result of equation *i*.

Assume that *M*_*i*_ nondominated solutions are obtained for the *i*th equation. A popular aggregation method is to compute the linear aggregation sum (LAS):

(9) LScorei,kλ=λScore1i,k+1−λScore2i,k,k=1,…,Mi

for a given weight *λ* ∈ [0,1] and select one solution with minimum sum as the inference result of equation *i*. However, the LAS method, which is dedicated to compare the difference of sum between all configurations, focuses on the most acute decrease of sum as connection number increases. As a result, a “too sparse” network is obtained.

To address the merit of LAS method, we would like to take the aggregation product (AP)

(10) $$\begin{array}{c} PScore_{i,k}\left(\;\lambda \;\right)=Score1_{i,k}^{\lambda }\cdot Score2_{i,k}^{1-\lambda } \end{array}$$

as the criterion of result confirmation. For a given weight *λ* ∈ [0,1], we select the *k*_*i*_th solution with

(11) $$\begin{array}{c} PScore_{i,k_{i}}\left(\;\lambda \;\right)=\underset{k=1,\ldots ,M_{i}}{\min}PScore_{i,k}\left(\;\lambda \;\right) \end{array}$$

as the inference result of equation *i*. Combining inference results of all equations, we then get the overall inference result of the investigated S-System and evaluate its quality by numbers of the true positives (TPs), false positives (FPs), true negatives (TNs), and false positives (FPs). With change of parameter *λ*, the Receiver Operator Characteristic (ROC) curve is taken as the illustration of quality for the nondominated solutions of (6) obtained by the mv-MOEA.

### 2.4. The Automatic Procedure for Selecting the Final Inference Results from the Obtained Nondominated Solutions

Although we can get an inference result for a given *λ*, we do not know which value of *λ* corresponds to the “right” tradeoff between two objectives of (6). In this work, we would like to propose an automatic selection procedure (ASP) for confirmation of the final inference result. When an inference result of the investigated S-System is obtained for a given weight *λ*, we can simultaneously get a sum vector as

(12) $$\begin{array}{c} Vec\left(\;\lambda \;\right)=\left(\;\overset{N}{\underset{i=1}{\sum }}S1\left(\;i,\lambda \;\right),\overset{N}{\underset{i=1}{\sum }}S2\left(\;i,\lambda \;\right)\;\right), \end{array}$$

where *S*1(*i*, *λ*) = Score1_*i*,*k*_*i*__ and *S*2(*i*, *λ*) = Score2_*i*,*k*_*i*__ are the respective normalized objective values of the inference result for equation *i*. When sampling *λ* in [0,1], we also get a collection of sum vectors constituting a normalized Pareto front in the objective space.

Since there are only two objectives to be considered, improvement of one objective will lead to deterioration of another. If small improvement of one objective results in a severe deterioration of another, the solutions constitute the so-called knee regions. According to the method proposed in [26], Li et al. suggest to locate the knee region to select one result from the Pareto front, where the angle-based method [27] is employed to seek the knee points on the combined Pareto front. In this method, two adjacent points are incorporated to compute the tradeoff angle of a point, and one with maximum tradeoff angle is selected as the preferred sum vector. Recall the value of *λ* corresponding to this sum vector; we can get the final inference results of all equations via (10) and (11) and combining them to get the overall inference result of the investigated S-System.

## 3. Results and Discussions

In this section, performances of the IM-MOEO are validated by two investigated S-Systems. To demonstrate the inference precision and the robustness of our method, we first infer an artificial network via noise-free and noisy simulated data. Then, the Ethanol Production System by Yeast is investigated to show how our method obtains a sparse network by focusing on simulation of its dynamical property. When generating simulated data for two problems, we sample 15 time points uniformly in the same time intervals as those investigated in the literatures for comparison.

### 3.1. Inference of an Artificial System by Noise-Free and Noisy Data

The investigated artificial network S1 is an artificial S-System illustrated in Table 1. Some previous researches [15, 20, 28–32] have been performed on this network to demonstrate efficiencies of S-System inference methods. Thus, we also reconstruct it to validate competitiveness of our method.

#### 3.1.1. Experimental Settings

To perform a fair comparison with the method proposed in [15], noise-free data are time-course series generated by sampling at 15 time points for 4 diverse initial conditions. Search regions for the kinetic orders and rate constants are set as [−3,3] and [0,10], respectively. Due to the difficulty of multiobjective optimization, we employ a population of 100 individuals and report the obtained nondominated solutions after 2000 iterations. For each equation of this artificial system, the algorithm is independently run for 10 times to obtain a satisfactory inference result. The noise-free, 5%-noise, 15%-noise, and 25%-noise data are generated with the same method proposed in [15].

#### 3.1.2. Inference Results of S1

Performances of IM-MOEO versus those of some previous researches are first investigated by the true positive rate (TPR) and false positive rate (FPR) which are computed as

**Figure 1:**
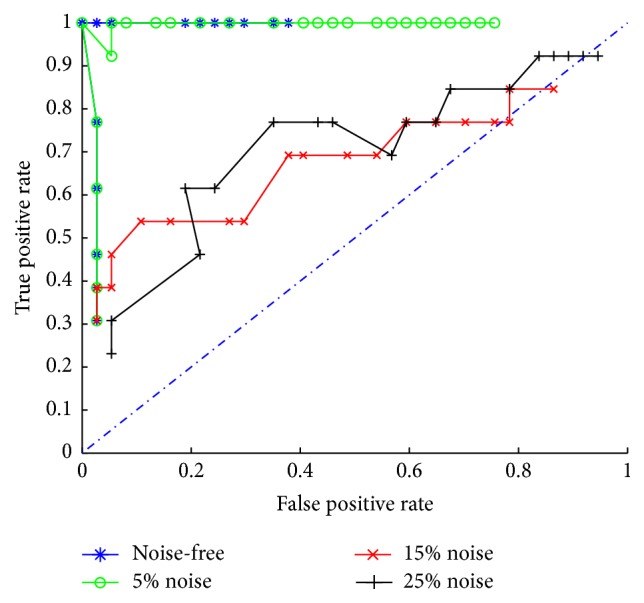
ROC plot of the inference results for the artificial network S1.

(13) TPR=TPTP+FN,FPR=FPTN+FP,

where TP, FN, TN, and FP represent the true positive (TP), false negative (FN), true negative (TN), and false positive (FP) predictions of the parameters. Then, the ROC curves are illustrated in Figure 1 for noise-free, 5%-noise, 15%-noise, and 25%-noise data. It is noted that the proposed method can generally identify the network via data of noise-free and 5% noise; however, its performance deteriorates quickly when the noise rate rises to 15%. It could be attributed to the inherent mechanism of our method, that is, incorporation of binary variables for identification of network connections. Since the *L*_0_ norm of parameter vectors to be minimized is confirmed by binary variables, the proposed MOEA is dedicated to search the sparse network topologies, which makes it less compatible with the data noise.

Meanwhile, this method is significantly insensitive to the parameter *λ*. Thanks to incorporation of the *L*_0_ norm instead of the *L*_1_ norm of the parameter vectors, the inference results are not sensitive to values of *λ*. As a result, 101 uniformly sampled values in [0,1] of *λ* only contribute to several different results of the artificial system. This suggests that if we can appropriately dispose of the difficulty introduced by incorporation of *L*_0_ norm in the optimization model, it could be much more likely to select a preferred nondominated result of the multiobjective optimization via the aggregation method represented by (10) and (11).

By locating the knee region of the objective curve, we can get the final reconstruction results of the artificial system S1. The results again demonstrate that our method is not sensitive to value of *λ*, because the knee points are, respectively, obtained for noise-free, 5% noise, 15% noise, and 25% noise data when *λ* varies in [0.31,0.68], [0.43,0.59], [0.49,0.65], and [0.40,0.54], respectively. For comparison with* L*1-DPSO [15], the obtained reconstruction results are included in Table 2 for four different data sets.

It is shown that both our method and* L*1-DPSO can obtain the correct network of S1 by noise-free data. Generally, our method can obtain more accurate parameter values for noise-free data, except that parameters of (3) are less precise. This is because concentration of the 3rd component quickly reaches its stationary state, and consequently, less information could be extracted by fitting the time-series data of derivatives.

When it comes to the noise data, the competitiveness of our method is highlighted by the inference results. Our method can always obtain sparse network topologies of S1, even if the noise rate (NR) varies from 5% to 25%. By contrast, it is a mission impossible in the method proposed in [15] to set a uniform threshold value for pruning of network connections, because increase of NR definitely increases fitting errors of the* L*1-DPSO, which further influences the inference result of the investigated S-System. Although the data noise also lowers the precision of our inference results, incorporation of the biobjective model and the automatic confirmation scheme ensures that a sparse network can always be obtained, and for most cases, sparseness (rate of true connections versus possible connections) of the obtained network is similar to the true network. The superiority is highlighted by the inference result obtained when NR of data is relatively low—our method can correctly identify the correct network topologies via 5%-noise data. Meanwhile, the automatic confirmation of sparse network also meets a problem—sparse network topology is sensitive to data noise. As a result, for 15%- and 25%-noise data, more network connections are wrongly identified by our method.

### 3.2. Identification of the Yeast Fermentation Pathway Dynamics

As an example of real biochemical networks, the yeast fermentation pathway proposed in [33] is also investigated in this work. Its S-System model contains five dependent variables: glucose (*X*_1_), glucose-6-phosphate (*X*_2_), fructose-1,6-diphosphate (*X*_3_), phosphoenolpyruvate (*X*_4_), and ATP (*X*_5_) and eight independent variables with the following steady-state values: glucose uptake (*X*_6_; 47.5 mM/min), hexokinase (*X*_7_; 24.1 mM/min), phosphofructokinase (*X*_8_; 53.9 mM/min), glyceraldehyde 3-phosphate dehydrogenase (*X*_9_; 91.4 mM/min), pyruvate kinase (*X*_10_; 18.1 mM/min), polysaccharide storage (*X*_11_; 82.9 mM/min), glycerol production (*X*_12_; 92.4 mM/min), and ATPase (*X*_13_; 1.0 mM/min) [34]. So, the S-System S2 is

(14) dX1dt=1.0006X2−0.0492X6−1.6497X10.5582X50.0456X7,dX2dt=1.6497X10.5582X50.0465X7−0.5793X20.5097X5−0.2218X80.8322X110.1678,dX3dt=0.4536X20.4407X5−0.2665X8−0.2456X30.4506X40.0441X50.092X90.8547X120.1453,dX4dt=0.2365X30.5285X50.0994X9−2.0892X3−0.0075X40.304X50.0484X10,dX5dt=1.406X30.2605X40.152X50.0739X90.5X100.5−2.9437X10.1962X20.1791X50.2354X70.3514X80.2925X110.0589X130.297.

It is inferred via a time-course data generated by 10 random initial concentrations, with each initial condition, that 15 time points are sampled. The search ranges of the parameters are [0.0,3.0] for *α*_*i*_ and *β*_*i*_ and [−1.0,1.0] for *g*_*ij*_ and *h*_*ij*_. Employing the mv-MOEA solving model (6), we can obtain a collection of configurations (nondominated solutions) for each equation. Then, the ROC curve could be obtained by sampling *λ* in [0,1]. Finally, the automatic selection strategy is implemented obtaining its final inference result.

**Figure 2:**
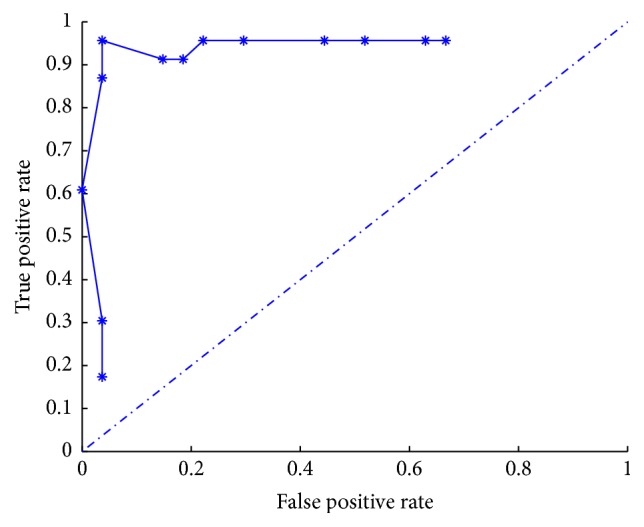
ROC plot of the inference results for the real network S2.

Although there are several weak connections in the system S2, the mv-MOEA can get the correct network topologies for all equations (it means that, for each equation, the mv-MOEA can obtain one nondominated solution indicating the correct network topology). To demonstrate a highlighted illustration of the nondominated solutions obtained by mv-MOEA, we only illustrate in Figure 2 the ROC curve obtained by sampling *λ* in [0,1]. The ROC curve demonstrates a satisfactory result for inference of S2, which shows that the biobjective optimization model (6) and mv-MOEA can work well for this network. What should be noted is that the ROC curve does not include the point (0,1), despite the fact that the correct topology corresponding to each equation is included in the obtained collections of nondominated solutions. The reason is that for all equations the correct topologies corresponding to various network connections and fitting errors, a uniform setting of *λ* possibly does not locate all correct configurations at the same time. Consequently, the overall inference result of S2 does not locate all correct network topologies for all equations.

**Figure 3:**
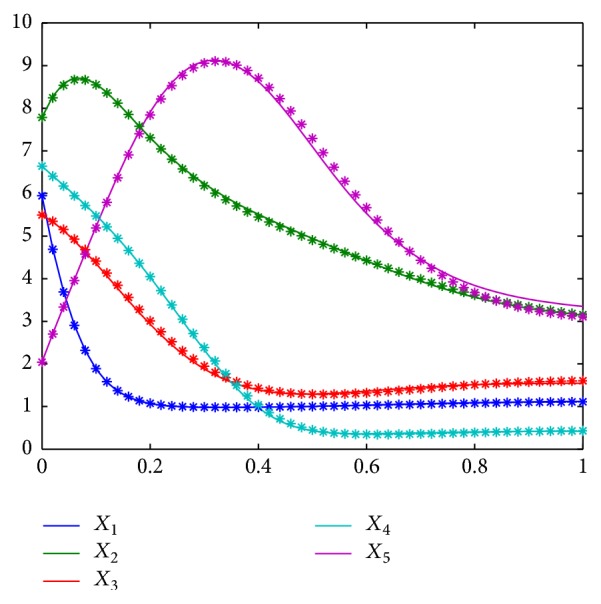
Dynamic curves of the obtained results for bf S2.

By employing the ASP on the obtained nondominated solutions, we get the final inference result of system S2 included in Table 3. Just for the same reason, the final result obtained by the ASP has got a false connection and missed several weak connections. To evaluate how these wrongly identified connections influence dynamical properties of the investigated S-System, we compare the dynamical curves of all components in one plot illustrated in Figure 3. It is demonstrated that the dynamical properties of obtained network are almost consistent to that of the true network, even if several connections are wrongly identified. Premised on the result of data fitting, IM-MOEO is dedicated to obtain a sparse network. Because all the wrong identifications are weak connections that do not significantly influence the dynamic properties of this network, these weak connections are not correctly confirmed when the ASP gets a tradeoff between data fitting error and network sparseness. As a result, it comes to a sparse network that has a low FPR and a high TPR.

## 4. Conclusion and Discussion

To address defects of existing inference methods, we propose a biobjective optimization model for identification of S-Systems, an efficient mix-variable multiobjective evolutionary algorithm to solve the biobjective model, and an automatic selection scheme for confirmation of the final inference result. Although introduction of binary variables and *L*_0_-norm make the biobjective optimization model harder to solve, the proposed mv-MOEA can deal with it with satisfactory performances. The automatic selection scheme demonstrates to be intelligent in investigated benchmark networks; however, it sometimes misses some weak connections due to its preference to sparse network topologies. In general, the biobjective optimization method accompanied with the automatic selection scheme is a universal method for inference of BSs, because the biobjective model could be applied to BSs of any size, and no problem-dependent parameters are needed for its successful implement. By inferring two widely investigated small-scale networks, we do validate its effectiveness compared with previous researches. However, when applied to large-scale BSs, mv-MOEA could be computationally expensive and perform unsatisfactorily attributed to data noise as well as lack of sufficient samples. Thus, data mining methods should be incorporated to boost its applications on large-scale BSs. Further improvement of this method could be focused on enhancement of its performance on noise data and its applications on large-scale BSs.

## Figures and Tables

**Table 1:** Parameter values of the artificial network S1.

*i*	*α*_*i*_	*g*_*i*1_	*g*_*i*2_	*g*_*i*3_	*g*_*i*4_	*g*_*i*5_	*β*_*i*_	*h*_*i*1_	*h*_*i*2_	*h*_*i*3_	*h*_*i*4_	*h*_*i*5_
1	5.0	0.0	0.0	1.0	0.0	−1.0	10.0	2.0	0.0	0.0	0.0	0.0
2	10.0	2.0	0.0	0.0	0.0	0.0	10.0	0.0	2.0	0.0	0.0	0.0
3	10.0	0.0	−1.0	0.0	0.0	0.0	10.0	0.0	−1.0	2.0	0.0	0.0
4	8.0	0.0	0.0	2.0	0.0	−1.0	10.0	0.0	0.0	0.0	2.0	0.0
5	10.0	0.0	0.0	0.0	2.0	0.0	10.0	0.0	0.0	0.0	0.0	2.0

**Table 2:** Comparison between IM-MOEO and *L*1-DPSO for the artificial network S1.

NR	*i*	Method	*α*_*i*_	*g*_*i*1_	*g*_*i*2_	*g*_*i*3_	*g*_*i*4_	*g*_*i*5_	*β*_*i*_	*h*_*i*1_	*h*_*i*2_	*h*_*i*3_	*h*_*i*4_	*h*_*i*5_
Free	1	IM-MOEO	4.928	0.0	0.0	0.996	0.0	−1.008	9.908	2.026	0.0	0.0	0.0	0.0
		*L*1-DPSO	4.387	0.0	0.0	1.425	0.0	−0.896	9.567	1.465	0.0	0.0	0.0	0.0
	2	IM-MOEO	9.985	2.002	0.0	0.0	0.0	0.0	9.983	0.0	2.003	0.0	0.0	0.0
		*L*1-DPSO	9.324	1.789	0.0	0.0	0.0	0.0	10.562	0.0	2.058	0.0	0.0	0.0
	3	IM-MOEO	5.381	0.0	−1.309	0.0	0.0	0.0	4.040	0.0	−1.459	1.984	0.0	0.0
		*L*1-DPSO	10.879	0.0	−1.659	0.0	0.0	0.0	9.847	0.0	−1.245	1.875	0.0	0.0
	4	IM-MOEO	7.966	0.0	0.0	2.023	0.0	−1.007	9.993	0.0	0.0	0.0	1.952	0.0
		*L*1-DPSO	7.795	0.0	0.0	2.054	0.0	−1.021	9.739	0.0	0.0	0.0	1.975	0.0
	5	IM-MOEO	9.962	0.0	0.0	0.0	2.005	0.0	9.967	0.0	0.0	0.0	0.0	2.014
		*L*1-DPSO	9.632	0.0	0.0	0.0	2.056	0.0	9.567	0.0	0.0	0.0	0.0	2.136

5%	1	IM-MOEO	5.417	0.0	0.0	0.893	0.0	−0.1.011	9.834	1.527	0.0	0.0	0.0	0.0
		*L*1-DPSO	4.332	−0.070	−0.098	1.783	0.070	−0.568	10.235	1.235	−0.030	0.138	0.042	0.027
	2	IM-MOEO	8.376	1.635	0.0	0.0	0.0	0.0	8.966	0.0	2.609	−0.674	0.0	0.0
		*L*1-DPSO	9.299	1.549	−0.089	−0.025	−0.139	−0.139	11.263	−0.133	2.432	0.048	−0.038	−0.150
	3	IM-MOEO	1.234	0.0	−0.690	−0.3555	−0.886	0.0	1.137	0.0	0.0	0.0	0.0	0.0
		*L*1-DPSO	10.771	0.086	−2.568	0.003	−0.145	0.010	9.076	−0.101	−1.569	2.564	−0.092	−0.062
	4	IM-MOEO	7.513	0.0	0.0	1.379	0.0	−1.059	9.953	0.0	0.0	0.0	1.624	0.0
		*L*1-DPSO	8.214	−0.144	−0.092	2.785	−0.048	−0.626	9.671	0.032	0.148	−0.087	2.568	0.039
	5	IM-MOEO	10.00	0.0	0.0	0.0	1.686	0.0	8.316	0.0	0.0	0.0	0.0	1.586
		*L*1-DPSO	9.490	0.135	0.090	0.081	1.865	0.101	11.167	0.142	0.118	0.023	0.040	2.461

15%	1	IM-MOEO	4.355	0.0	0.0	0.619	0.0	−1.063	9.960	1.791	0	0	0	0
		*L*1-DPSO	3.987	−0.137	−0.432	2.654	0.426	−1.986	8.987	1.869	−0.086	−0.046	−0.137	0.078
	2	IM-MOEO	3.535	2.057	−0.779	0	0	0	0.00	0	0	0	0	0
		*L*1-DPSO	11.786	3.123	0.405	0.218	0.237	−0.258	11.786	−0.208	3.215	0.415	0.310	−0.461
	3	IM-MOEO	2.481	0.821	0.0	0.0	−2.379	0	1.737	0	0	0.0	0	0
		*L*1-DPSO	11.126	−0.020	−3.126	0.083	0.011	0.207	11.126	−0.244	−1.986	3.412	0.475	−0.369
	4	IM-MOEO	5.779	0.0	0.0	1.897	−0.839	−0.942	6.983	0.0	0.0	0.0	0.0	0.0
		*L*1-DPSO	6.976	0.0	0.0	0.0	1.116	−0.646	4.722	0.0	0.0	0.0	0.0	0.0
	5	IM-MOEO	6.976	0.0	0.0	0.0	1.116	−0.646	4.722	0.0	0.0	0.0	0.0	0.0
		*L*1-DPSO	8.334	0.208	0.357	−0.327	3.214	0.295	8.334	−0.038	−0.428	−0.318	0.282	3.604

25%	1	IM-MOEO	8.150	−0.272	0.0	0.645	0.0	−0.495	8.089	0.0	0.0	0.0	0.0	0.0
		*L*1-DPSO	7.894	0.481	−0.759	3.879	0.946	−2.963	19.235	4.651	−0.326	0.356	0.738	0.526
	2	IM-MOEO	10.00	0.8838	−0.2913	0.0	0.0	0.0	6.087	0.0	0.0	0.0	0.0	0.0
		*L*1-DPSO	15.031	4.129	0.110	−0.491	−0.557	−0.423	18.605	−0.215	4.152	0.627	0.494	−0.723
	3	IM-MOEO	6.689	0.0	−0.257	−0.909	0.0	0.0	6.336	0.0	0.0	0.0	0.0	0.0
		*L*1-DPSO	18.678	0.859	−4.214	−0.388	0.305	−0.042	15.065	−0.581	−2.546	4.421	−0.170	0.556
	4	IM-MOEO	7.2311	0.0	−0.290	0.0	0.0	−0.681	9.319	0.0	0.0	−0.591	0.0	0
		*L*1-DPSO	6.579	−0.215	0.199	4.568	−0.635	−2.655	17.036	0.250	0.859	0.258	5.123	0.190
	5	IM-MOEO	6.773	0.0	0.0	0.0	0.0	0.0	1.342	0.0	0.825	0.0	−1.415	0.0
		*L*1-DPSO	6.598	−0.941	0.487	−0.833	4.125	−0.564	14.905	0.060	−0.106	0.785	−0.349	4.843

**Table 3:** Reconstruction result of IM-MOEO for the network S2.

*i*	*α*_*i*_	*g*_*i*1_	*g*_*i*2_	*g*_*i*3_	*g*_*i*4_	*g*_*i*5_	*β*_*i*_	*h*_*i*1_	*h*_*i*2_	*h*_*i*3_	*h*_*i*4_	*h*_*i*5_
1	0.9009	0	−0.0495	0	0	0	1.4985	0.5970	0	0	0	0.0427
2	1.1871	0.5822	0	0	0	0.1835	0.3927	0	0.5368	0	0	−0.0732
3	0.5014	0	0.2768	0	0	0	0.2515	0	0	0.3901	0	0.2565
4	0.1935	0	0	0.5777	0	0.0599	1.9370	0	0	−0.0779	0.3684	0
5	0.8562	0	0	0.3766	0.1770	0.0581	1.5422	0.2863	0.2728	0	−0.0541	0.2691
